# Full-parameter omnidirectional transformation optical devices

**DOI:** 10.1093/nsr/nwad171

**Published:** 2023-06-08

**Authors:** Yuan Gao, Yu Luo, Jingjing Zhang, Zhengjie Huang, Bin Zheng, Hongsheng Chen, Dexin Ye

**Affiliations:** Interdisciplinary Center for Quantum Information, State Key Laboratory of Extreme Photonics and Instrumentation, ZJU-Hangzhou Global Scientific and Technological Innovation Center, Zhejiang University, Hangzhou 310027, China; School of Electrical and Electronic Engineering, Nanyang Technological University, Singapore 639798, Singapore; CNRS-International-NTU-Thales Research Alliance, Nanyang Technological University, Singapore 637553, Singapore; Institute of Electromagnetic Space, Southeast University, Nanjing 210096, China; State Key Laboratory of Millimeter Waves, Southeast University, Nanjing 210096, China; Interdisciplinary Center for Quantum Information, State Key Laboratory of Extreme Photonics and Instrumentation, ZJU-Hangzhou Global Scientific and Technological Innovation Center, Zhejiang University, Hangzhou 310027, China; Interdisciplinary Center for Quantum Information, State Key Laboratory of Extreme Photonics and Instrumentation, ZJU-Hangzhou Global Scientific and Technological Innovation Center, Zhejiang University, Hangzhou 310027, China; International Joint Innovation Center, The Electromagnetics Academy at Zhejiang University, Zhejiang University, Haining 314400, China; Key Laboratory of Advanced Micro/Nano Electronic Devices & Smart Systems of Zhejiang, Jinhua Institute of Zhejiang University, Zhejiang University, Jinhua 321099, China; Shaoxing Institute of Zhejiang University, Zhejiang University, Shaoxing 312000, China; Interdisciplinary Center for Quantum Information, State Key Laboratory of Extreme Photonics and Instrumentation, ZJU-Hangzhou Global Scientific and Technological Innovation Center, Zhejiang University, Hangzhou 310027, China; International Joint Innovation Center, The Electromagnetics Academy at Zhejiang University, Zhejiang University, Haining 314400, China; Key Laboratory of Advanced Micro/Nano Electronic Devices & Smart Systems of Zhejiang, Jinhua Institute of Zhejiang University, Zhejiang University, Jinhua 321099, China; Shaoxing Institute of Zhejiang University, Zhejiang University, Shaoxing 312000, China; Interdisciplinary Center for Quantum Information, State Key Laboratory of Extreme Photonics and Instrumentation, ZJU-Hangzhou Global Scientific and Technological Innovation Center, Zhejiang University, Hangzhou 310027, China

**Keywords:** transformation optical devices, omnidirectional impedance matching, full-parameter invisibility cloak

## Abstract

Transformation optics (TO) provides an unprecedented technique to control electromagnetic (EM) waves by engineering the constitutive parameters of the surrounding medium through a proper spatial transformation. In general, ideal transformation optical devices require simultaneous electric and magnetic responses along all three dimensions. To ease the practical implementation, previous studies usually made use of reduced parameters or other simplified approaches, which inevitably introduce extra reflection or unwanted phase shift. Up to today, experimental realizations of full-parameter transformation optical devices in free space are still quite limited. Here, a general design strategy is proposed to solve this problem. As a specific example, a full-parameter spatial-compression TO medium with constitutive parameters taking the diagonal form diag(*a, a*, 1/*a*) for the TM wave incidence was designed and realized experimentally. Such spatial-compression TO media were then applied to the implementation of an ideal omnidirectional invisibility cloak capable of concealing a large-scale object over a wide range of illumination angles. Both the simulation and experiment confirm that the cloak allows for nearly unity transmission of EM waves in the forward direction without introducing extra scattering or phase shift. This work constitutes an important stepping stone for future practical implementation of arbitrary full-parameter omnidirectional transformation optical devices.

## Teaser

Using a judiciously designed full-parameter transformation optical metamaterial, an ideal omnidirectional invisibility cloak capable of concealing a large-scale object in the free space is implemented experimentally.

## INTRODUCTION

Transformation optics (TO) [1] offers excellent versatility for manipulating electromagnetic (EM) waves using subtly devised materials. The past decade has witnessed the rapid development of TO, through which various novel optical devices such as invisibility cloaks [2–17], concentrators [18], flattened Luneberg lenses [19] and sharp waveguide bending [20,21] have been designed and realized experimentally. In general, the constitutive parameters of ideal transformation optical devices (e.g. the invisibility cloak) are spatially inhomogeneous and often have singular values [3,18], making the practical implementations difficult. Although the simplified designs [4,7–9,11,12] based on the eikonal approximation can avoid the material singularity and maintain the trajectory of light in the transformation optical device, unwanted reflection is introduced at the interface between the transformation optical device and the background medium. In other words, the simplified designs sacrifice the impedance matching and deteriorate the transparent properties of the transformation optical devices. In 2013, Landy *et al.* fabricated a full-parameter unidirectional metamaterial cloak [14] beyond the eikonal approximations. However, to avoid singular parameters, this cloak works only for one particular incident angle. Moreover, it was implemented in a parallel-plate waveguide (not in free space) in order to achieve simultaneous electric and magnetic responses required by the ideal TO medium. To realize omnidirectional transparency in free space, several strategies have been proposed. For example, in 2016, Luo *et al.* realized the perfectly transparent media with omnidirectional impedance matching using the spatially dispersive effective parameters of photonic crystals [22]. Another approach based on the angle-independent longitudinal impedance matching through a judiciously engineered metasurface [23–25] can also achieve full transparency over a wide range of incident angles. The recently proposed transformation-invariant metamaterials [26] and Fabry-Pérot meta-coating [27] can realize all-angle impedance matching. Nevertheless, all relevant experimental realizations either suffer from strong phase shifts (leading to a lateral shift from the transmitted light beam with respect to the incident one) or work only for a specific waveguide mode (making the design inappropriate for free-space implementations). Up to today, there lacks a general approach to implement full-parameter omnidirectional transformation optical devices without any phase shifts.

In this paper, we present a solution to this problem by designing a three-dimensional (3D) metamaterial with the relative permittivity and permeability judiciously optimized to take the diagonal form diag(*a, a*, 1/*a*) for the TM wave incidence, where *a* can be an arbitrary constant. Such constitutive parameters satisfy the condition of uniaxial perfectly matched layer [28], thereby providing us a stepping stone to implement various full-parameter transformation optical devices. As a specific example, we apply this metamaterial to the realization of a full-parameter omnidirectional cloak in free space. Both full-wave numerical simulation and microwave experiment confirm that this cloak can conceal a large-scale object for light incident from any direction without introducing any phase shift.

## THEORETICAL ANALYSIS

The 3D metamaterial key to our realization is also known as the spatial-compression TO medium, whose constitutive parameters are obtained by compressing a slab along the direction perpendicular to its surface:


\begin{equation*}
\begin{array}{l@{}l@{\quad}} z^{\prime} = \left\{ {\begin{array}{l@{}c@{}}
{z - {z}_{1} + {z^{\prime}_{1}}} &\quad z < {z}_{1}\\
{z^{\prime}_{1}} + \frac{{z^{\prime}_{2}} - {z^{\prime}_{1}}} {{{z}_{2} - {z}_{1}}}\left( {z - {z}_{1}} \right)&\quad {z}_{1} \le z < {z}_{2}\\
{z - {z}_{2} + {z^{\prime}_{2}}} &\quad z \ge {z}_{2}
\end{array}} \right.\\
x^{\prime} = x\\
y^{\prime} = y \qquad\qquad\qquad\qquad\qquad\qquad\qquad\quad\,\, (1)
\end{array}.
\end{equation*}

**Figure 1.**
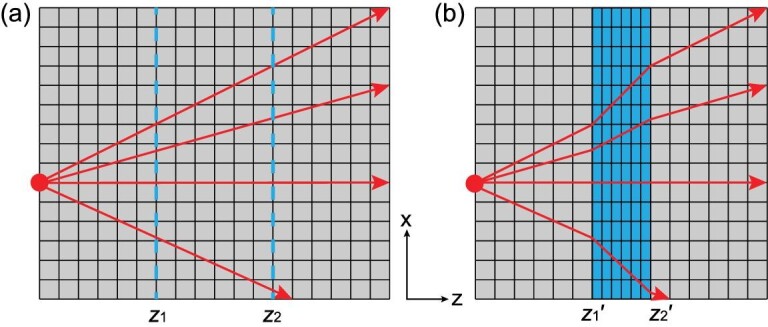
Coordinate transformation to compress a space along the *z*-axis. The initial space (a) and the physical space (b). The red arrows represent the light rays from a point source, and the light follows a distorted trajectory without any reflection when passing through the blue compressed region.

The schematic of such a transformation is given in Fig. 1, where a slab located between *z*_1_*_ _≤ z ≤ z*_2_ (Fig. 1a) in the initial space is transformed into another slab located between *z^’^*_1 _*≤ z ≤ z^’^*_2_ (Fig. 1b) in the physical space. Since we are interested in designing transformation optical devices in free space, the relative permittivity and permeability of the initial slab are assumed to be 1, and according to theory of TO, the material tensors of the transformed slab take the uniaxial form [29]


(2) \begin{equation*}
{\varepsilon}^{\prime^{\leftrightarrow}} = {\mu}^{\prime^{\leftrightarrow}} = {\rm diag}\left(a,a,\frac{1}{a}\right),
\end{equation*}

where *a* = (*z*_2_ − *z*_1_)/(*z*′_2_ − *z*′_1_) is the compression factor, i.e. *a *> 1 indicates that the space is compressed along the *z*-axis while *a *< 1 implies that the space is stretched. It should be noted that such a kind of media allows for the total transmission of the incident wave without any reflection at arbitrary incident angles [28].

## DESIGN AND SIMULATION

**Figure 2.**
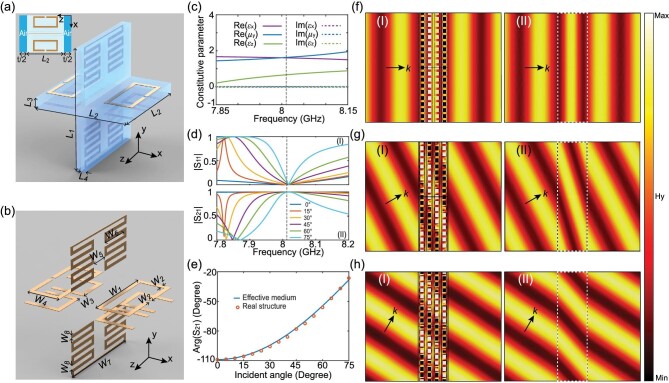
Design of a full-parameter spatial-compression TO metamaterial. (a) Unit cell of the designed TO metamaterial. Here, *L*_1_ = 5.6 mm, *L*_2_ = 5 mm, *L*_3_ = 0.5 mm, *L*_4_ = 0.25 mm, and *t*/2 = 1 mm. The inset shows the top view of the unit cell in the simulation. (b) Metallic structures in the unit cell, where *W*_1_ = 3.03 mm, *W*_2 _= 0.2 mm, *W*_3_ = 0.3 mm, *W*_4 _= 1.515 mm, *W*_5 _= 0.84 mm, *W*_6_ = 1.68 mm, *W*_7 _= 4.2 mm, and *W*_8 _= 0.15 mm. (c) Retrieved effective constitutive parameters for one single layer of the designed TO metamaterial. (d) Simulated reflection (|S_11_|, I) and transmission (|S_21_|, II) at different incident angles. (e) Simulated transmission phase of the designed TO metamaterial (yellow dots) at 8.012 GHz and the calculated one (blue line) using the effective constitutive parameters. Simulated magnetic field distributions at 8.012 GHz around four-layer metamaterial structures (I) and the effective media (II) when the incident angle is (f) 0°, (g) 30°, and (h) 60°.

In this section, we present a design strategy based on 3D metamaterials to implement a full-parameter TO medium at the microwave band for the TM wave incidence. The unit cell comprises two orthogonal split-ring resonators (SRRs) printed on two sides of a dielectric substrate in the *x*–*z* plane, as shown in Fig. 2a. For the TM wave incidence with a *y*- polarized magnetic field, a magnetic resonance is induced and the effective relative permeability (*μ_y_*) larger than unity occurs below the resonance frequency [30]. Meanwhile, due to the presence of the dielectric substrate, the effective relative permittivity along the *x*-axis (*ϵ_x_*) is also larger than unity. In other words, through a judicious design, we can modulate the magnetic resonance to achieve *μ_y_* = *ϵ_x_* = *a *> 1 at any desired frequency. To engineer the permittivity along the *z*- axis to be *ϵ_z__ _****= ***1/*a*, an I-shaped electric resonator is inserted between two identical dielectric substrates. Such a structure has been widely used to obtain metamaterials with near-zero or negative permittivities [31]. Note that the symmetry of the unit cell eliminates the unwanted bianisotropic effect. A full-parameter TO medium is then obtained by periodically arranging the unit cells closely along the *x* and *y* directions.

Using the CST Microwave Studio^TM^, we performed full-wave simulations and optimized the EM responses. In the simulation, periodic boundary conditions are used in the *x* and *y* directions, and the incident magnetic field is polarized along the *y*-axis. The metallic structures are made by perfect electric conductor with a thickness of 0.018 mm, and the dielectric substrate (F4B with a relative permittivity of 3) is set to be lossless. Meanwhile, we introduced an air space with a thickness of *t*/2 on each side of the unit cell along the *z* direction, as shown in the inset of Fig. 2a. This air space makes the coupling between two adjacent layers small enough when we stack multiple layers into a bulk medium to implement complex transformation optical devices. Finally, a full-parameter spatial-compression TO metamaterial working around 8 GHz is obtained and the detailed geometry dimensions are given in the captions of Fig. 2a and 2b. Utilizing the extraction algorithm for the oblique incidence [32], we retrieved the three effective constitutive parameters. As shown in Fig. 2c, *μ_y_* = *ϵ_x_* = 1.61 = 1/*ϵ_z_* at 8.012 GHz, satisfying the condition of full-parameter TO media for the TM wave incidence.

To analyze its performance at the oblique incidence, we plot the simulated reflection (|S_11_|) and transmission (|S_21_|) of one single layer at different incident angles in Fig. 2d. Apparently, the reflection is smaller than 0.08 at 8.012 GHz, and the transmission is larger than 0.99, over a wide range of incident angles (0°–75°). Moreover, Fig. 2e depicts the simulated transmission phases of the designed metamaterial (yellow dots) and the calculated ones using the effective constitutive parameters (blue line). The excellent agreement between the real-structure simulation and the effective-medium calculation demonstrates the validity of the designed metamaterial in realizing full-parameter transformation optical devices.

To study the performance of the designed metamaterial in the bulk, we arrange four layers of unit cells into a bulk structure. The simulated magnetic field distributions at different incident angles (i.e. 0°, 30° and 60°) are plotted in panels (I) of Fig. 2f, 2g, and 2h, respectively. Apparently, light transmitted through the four-layer metamaterial structures continues propagating along the original direction without causing any obvious reflection. For comparison, we also plot, in panels (II) of Fig. 2f, 2g, and 2h, the simulated magnetic field distributions by replacing the real metamaterial structure with an effective medium characterized by *μ_y_* = *ϵ_x_* = 1.61 = 1/*ϵ_z_*. The magnetic field distributions in panels (I) and (II) are nearly identical, confirming that the designed bulk metamaterial can be applied to the realization of full-parameter transformation optical devices of arbitrary thicknesses.

## RESULTS

**Figure 3.**
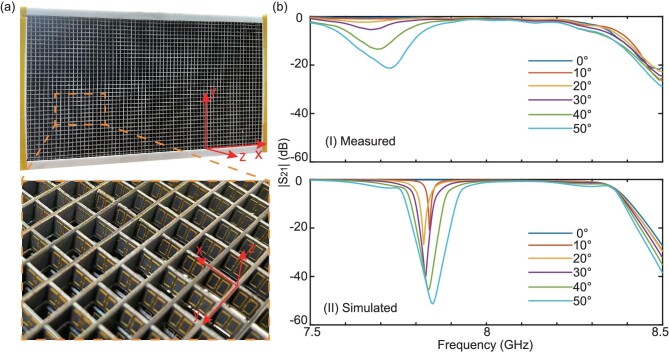
Implementation of the spatial-compression TO metamaterial. (a) Photograph of the fabricated four-layer metamaterial sample. (b) Measured (I) and simulated (II) transmission spectra of the four-layer metamaterial for different incident angles.

The measured transmission spectra at different incident angles are shown in panel (I) of Fig. 3b. We can see that under the normal incidence, the transmission coefficient reaches −0.6 dB at 8.005 GHz. It decreases slowly as the incident angle increases, but always stays larger than −1.5 dB for the incident angles ranging from 0° to 50°. For comparison, we also plot the simulated transmission spectra of the metamaterial in panel (II), where all the material losses are considered. In the simulation, the metal is set to be copper with a conductance of 5.986 × 10^7^ S/m and the loss tangent of F4B is 0.003. At 8.012 GHz, the simulated transmission coefficient decreases from −0.23 dB to −1.21 dB when the incident angle varies from 0° to 50°. The measured and simulated results agree well with each other despite of slight deviations in the transmission dips around 7.8 GHz. It should be noted that the material losses inevitably degrade the performance, due to that the operating frequency is close to the resonant frequency of the unit cell.

**Figure 4.**
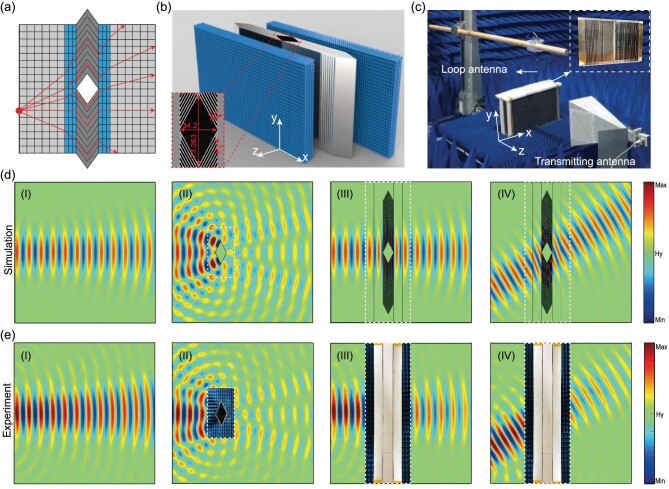
Design and measurements of an ideal omnidirectional invisibility cloak. (a) Illustration of the ideal omnidirectional invisibility cloak. (b) Schematic view of the ideal cloak based on the designed full-parameter spatial-compression TO metamaterial and the Fabry-Pérot layer. Inset: detailed dimensions of the Fabry-Pérot layer (unit: mm). (c) Experimental setup to measure the magnetic field distributions. Inset: The fabricated Fabry-Pérot layer. (d) Simulated magnetic field distributions at 8.012 GHz for air (I), the bare metal rhombus (II) and the designed cloak (III) under the normal incidence. Panel (IV) shows the result for the designed cloak under the oblique incidence (30°). (e) Measured magnetic field distributions at 8.005 GHz, which are corresponding to four cases shown in (d). The white dashed line rectangles in panels (II), (III), and (IV) represent the unmeasured regions, which are filled with the photographs of the fabricated samples.

Finally, we demonstrate experimentally that such a full-parameter spatial-compression TO metamaterial provides an excellent platform to realize ideal omnidirectional invisibility cloaks. Two recent works demonstrated that the scattering from a macroscopic object can be significantly suppressed by judiciously designed omnidirectional impedance-matching layers. One of them makes use of optic-null media [26] while the other relies on the Fabry-Pérot condition [27]. However, both approaches suffer from the wave displacement problem, i.e. the trajectory of the transmitted light is shifted along the transverse direction with respect to the incident one. Here, the wave displacement is compensated by our spatial-compression TO metamaterial, giving rise to a full-parameter omnidirectional cloak. As shown in Fig. 4a, our cloak is designed by combining the spatial-compression TO metamaterial (blue regions) with a Fabry-Pérot layer [27] (dark grey region). Similar to the optic-null medium, the intermediate Fabry-Pérot layer projects the input surface onto the output one of the device, and hence a wave displacement is introduced. To compensate this displacement, we use a spatial compression TO medium by setting the compression factor *a* = (T_1 _+ T_2_)/T_2_. Here, T_1_ denotes the thickness of the Fabry-Pérot layer and T_2_ denotes the thickness of the TO metamaterial, respectively. In such a case, the effective space of the whole transformation region exactly equates to the free space region with a thickness of T_1 _+ T_2_, and thus the displacement is eliminated.

The cloak is designed to work in air and the fabricated sample is given in Fig. 4b, where the inset depicts the detailed dimensions of the Fabry-Pérot layer. Considering that the total thickness (T_2_) of two TO metamaterial samples is 56 mm with *a* = 1.61, the thickness (T_1_) of the Fabry-Pérot layer is thus 34.2 mm, approximately the length of the smaller diagonal line of the rhombic cloaked region. Its larger diagonal line is set to be 73.26 mm in length, being around 1.96λ (λ is the operating wavelength in free space at 8.012 GHz).

For comparison, we plot, in Fig. 4d, the simulated magnetic field distributions at 8.012 GHz for air (I), the bare metal rhombus (II), the designed cloak under the normal incidence (III) and 30° oblique incidence (IV). In the simulation, the geometric dimensions of the Fabry-Pérot layer are the same with those shown in the inset of Fig. 4b, and the spatial-compression TO metamaterial is set to be homogeneous and lossless with *μ_y_* = *ϵ_x_* = 1.61 = 1/*ϵ_z_*. As expected, the metal rhombus strongly scatters the incident wave, and the designed cloak eliminates the scattering regardless of the incident angle of the TM wave.

Accordingly, panels (I)–(IV) of Fig. 4e display the measured results. The measured magnetic field distributions for air and the bare metal rhombus agree well with the simulated ones, confirming the effectiveness of the experimental setup. Panels (III) and (IV) of Fig. 4e show that the measured magnetic field distributions slightly deviate from the ones obtained by simulations due to the fabrication imperfections and material loss. Nevertheless, the incident TM waves can still circulate around the metal rhombus placed inside cloak without causing severe scattering or phase shift of the transmitted beam. The energy loss in the transmitted beam results from the resonance dissipation and impedance mismatch (owing to the fabrication imperfection and experimental misalignment) of the TO metamaterial samples. Consequently, the total transmission loss is about −1.2 dB, as shown in Fig. 3b.

**Figure 5.**
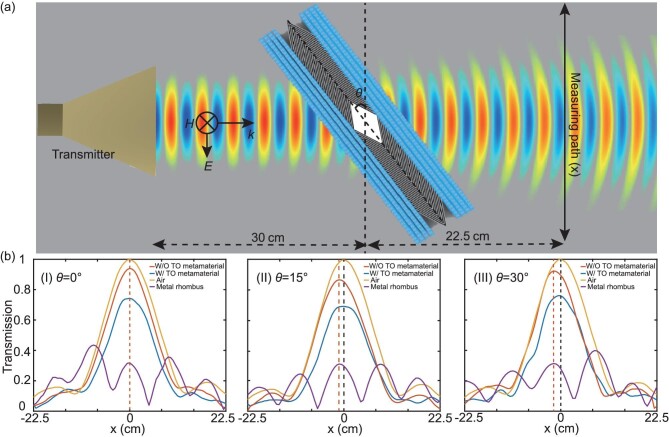
Transmission measurements. (a) Experimental setup for the transmission measurements along a line path (black solid line). (b) Measured transmission distributions at 8.005 GHz for four cases (Air, the bare metal rhombus, the Fabry-Pérot layer with and without the designed TO metamaterial) when the rotation angle *θ* is 0° (I), 15° (II), 30° (III).

To further illustrate that the spatial-compression medium can eliminate the phase shift of the transmitted beam, we measure the transmitted magnetic field amplitudes along a line shown in Fig. 5a. In the measurements, four cases are considered, i.e. air, the bare metal rhombus, the Fabry-Pérot layer with and without the spatial-compression medium. The measured amplitude distributions at 8.005 GHz with the rotation angle *θ* = 0°, 15°, 30°, are depicted in panels (I)–(III), respectively. Note that the results obtained are all normalized by the maximum transmission in the air background. We can see that the metal rhombus strongly disturbs the transmitted waves. The Fabry-Pérot layer without the spatial-compression medium can restore the wave patterns, however, the phase difference between the incident and transmitted waves shifts the trajectory of the transmitted beam, which increases for larger *θ*. Remarkably, the spatial-compression medium successfully compensates the phase shifts and eliminates the wave displacement.

## CONCLUSION

In conclusion, we demonstrated the physical implementation of a 3D omnidirectional full-parameter TO medium in free space background for the TM wave incidence. It is constructed by periodically arranging unit cells composed of SRRs and I-shaped electric resonators. The performance of the implemented medium is nearly independent on the layer number, providing us a stepping stone to realize various full-parameter transformation optical devices of arbitrary thicknesses. As a specific example, an omnidirectional full-parameter cloak that can conceal a large-scale object for arbitrary incident TM wave was fabricated and measured. Although fabrication imperfections and material loss inevitably degrade the performance, the theoretical analysis, full-wave simulations, and experimental measurements are agreeing well with each other. Relying on the resonant elements, the cloak experimentally realized in this work operates only over a narrow bandwidth. However, by combing with the approaches of dispersion engineering [33] and fast-light media [34], our design strategy can also be applied to full-parameter broadband TO devices.

In a recent work, Miller introduced the concept of overlapping nonlocality to estimate the minimum thickness of an optical device [35]. We highlight that the full-parameter design proposed in this work is barely limited by such a finite thickness constraint, whereas previous simplified designs are. For the full-parameter design, the point-to-point field mapping from the incident to the transmission side ensures that the overlapping nonlocality is close to zero, and conceptually, the thickness of the TO device can be made arbitrarily small if the compression factor a is large enough. On the contrary, diffractions induced by scattering (or reflection) from the simplified TO device result in nonzero overlapping nonlocality, which puts an ultimate finite thickness constraint on the TO device.

Although the cloak demonstrated here is planar and infinite in the transverse direction, a finite ideal omnidirectional invisibility cloak can also be designed using our approach, e.g. by replacing all phase compensating layers with our full-parameter compression TO metamaterial (see Supplementary Information). We expect our work will have immediate and far-reaching implications on practical applications of full-parameter transformation optical devices. Our design strategy can also be extended to other domains of physics, such as acoustics [36] and hydrodynamics [37].

## METHODS

### Sample fabrication

In the experiments, we fabricated two four-layer samples for the designed spatial-compression TO metamaterial using printed circuit board lithography, as shown in Fig. 3a. Each layer has a dimension of 340 × 212.8 × 5 mm^3^ (68 × 38 × 1 units) while each unit is fabricated according to the one designed in Fig. 2a and 2b.

In the fabrication of Fabry-Pérot layer, 56 bending iron sheets with height of 212.8 mm were used and aligned on a 3D-printed base. All other geometric dimensions are the same with those depicted in the inset of Fig. 4b.

### Experiments for measuring S-parameters

The transmission spectra of one sample were measured in a microwave anechoic chamber. A horn antenna was fed with a signal (from 7.5 GHz to 8.5 GHz) to excite the desired TM wave while a similar antenna was used to receive the signal and detect the transmissions (S_21_). The incident angle can be varied by rotating the sample. The measured transmission spectra at different incident angles were recorded by the vector network analyzer, as shown in panel (I) of Fig. 3b. Note that the transmission coefficients were all normalized by the ones measured by removing the sample.

### Experiments for scanning the field distributions

Figure 4c illustrates the experimental setup used to verify the performance of the cloak. A horn antenna filled with a homemade dielectric lens was used to excite the desired TM wave packet, with 270 mm away from the fabricated cloak. A small loop antenna with a radius of 2 mm, was utilized as the receiving antenna to detect the transmissions. In the measurements, the receiving antenna was fixed on a mechanical arm, which was mounted on a 3D measurement platform. The arm was programmed to move in the *x*–*z* plane to probe the local magnetic fields. The scanning area encompassed a 450 mm by 450 mm region and boasted a step resolution of 6 mm. It is noteworthy that this resolution is smaller than 1/6 of the wavelength, thus ensuring the recovered spatial field distributions has an exceptional quality.

## Supplementary Material

nwad171_Supplemental_File
