# Supplementary material for: Full-parameter omnidirectional transformation optical devices
Source: Natl Sci Rev. 2023 Jun 8;11(3):nwad171. doi: 10.1093/nsr/nwad171 (PMC10833459; doi:10.1093/nsr/nwad171)
Supplement: nwad171_Supplemental_File [file nwad171_supplemental_file.docx]

**Supplementary Information for**

**Full-parameter Omnidirectional Transformation Optical Devices**

Yuan Gao^1^, Yu Luo^2, 3*^, Jingjing Zhang^4, 5^, Zhengjie Huang^1^, Bin Zheng^1,6,7,8^,

Hongsheng Chen^1,6,7,8*^ and Dexin Ye^1*^

*^1^ Interdisciplinary Center for Quantum Information, State Key Laboratory of Extreme Photonics and Instrumentation, ZJU-Hangzhou Global Scientific and Technological Innovation Center, Zhejiang University, Hangzhou 310027, China.*

*^2^ School of Electrical and Electronic Engineering, Nanyang Technological University, 639798, Singapore*

*^3^ UMI 3288 CINTRA, CNRS/NTU/THALES, Nanyang Technological University, 50 Nanyang Drive, Singapore 637553, Singapore.*

*^4^ Institute of Electromagnetic Space, Southeast University, Nanjing 210096, China*

*^5^ State Key Laboratory of Millimeter Waves, Southeast University, Nanjing 210096, China*

*^6^ International Joint Innovation Center, The Electromagnetics Academy at Zhejiang University, Zhejiang University, Haining 314400, China*

*^7^Key Lab. of Advanced Micro/Nano Electronic Devices & Smart Systems of Zhejiang, Jinhua Institute of Zhejiang University, Zhejiang University, Jinhua 321099, China*

*^8^ Shaoxing Institute of Zhejiang University, Zhejiang University, Shaoxing 312000, China*

^*^*Corresponding authors. E-mail:* [*luoyu@ntu.edu.sg*](mailto:luoyu@ntu.edu.sg)*;* [*hansomchen@zju.edu.cn*](mailto:hansomchen@zju.edu.cn)*;* [*desy@zju.edu.cn*](mailto:desy@zju.edu.cn)

**Finite-sized omnidirectional invisibility cloak design based on the proposed full-parameter TO medium**

Although our experimental demonstration is performed using the infinite cloak design (an infinite planar cloak), the proposed spatial-compression TO metamaterial can also be used to achieve a more compact and conformal one, e.g., a finite-sized omnidirectional cloak.

Fig. S1(a) shows the coordinate transformation for such a finite-sized omnidirectional cloak. Here, a homogeneous transformation is adopted. In the virtual space (Top panel), a square of side 2*a* is divided into four identical isosceles right triangles, one of which is highlighted as green. Due to the symmetry, we only consider the green one in the coordinate transformation. This isosceles right triangle is compressed into a green isosceles triangle (Region I) of height *b* in the physical space (Bottom panel). Meanwhile, the orange edge of the triangle in the virtual space is stretched into an orange triangle (Region II) in the physical space. In such a case, the blue square (Region III) in the physical space is actually compressed into a blue point in the virtual space with zero cross section, i.e., an arbitrary object in Region III will be omnidirectionally invisible. According to the TO theory, we can get the constitutive parameters of region I and region II for the TM-polarized wave: *μ*^I^*_x_* = *ε*^I^*_y_* = 1/*ε*^I^*_z_* = *a*/*b*, *μ*^II^*_x_* = *ε*^II^*_y_* = 1/*ε*^II^*_z_* = 0. Apparently, the region II is the transformation-invariant metamaterial that can be realized by anisotropic photonic doping of epsilon-near-zero medium and perfect electric conductor [S1], and the region I is the proposed spatial-compression TO metamaterial.

As an example, we show the simulated results of such a cloak under the illumination of a point source in Fig.S1(b), which demonstrates an excellent cloaking effect. Here, *a*= 4λ, *b*= 4λ/1.61, where λ is the wavelength in the free space. In such a case, the region I is exactly with the designed TO metamaterial in this paper, satisfying *μ*^I^*_x_* = *ε*^I^*_y_* = 1/*ε*^I^*_z_* = 1.61.


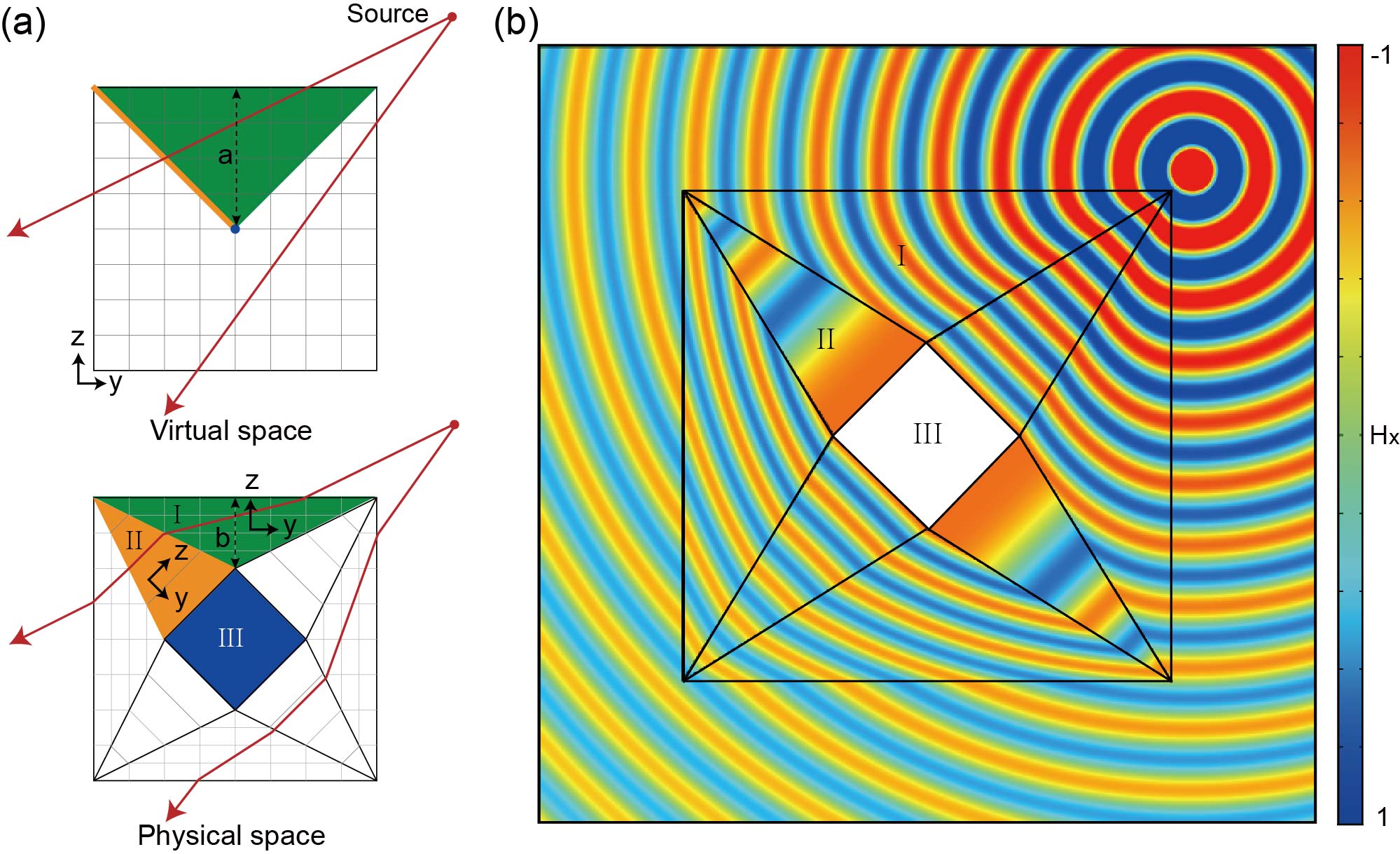


**Figure S1**. (a) The coordinate transformation for a finite-sized omnidirectional invisibility cloak design. (b) Simulated magnetic field distribution around the invisibility cloak under the illumination of a point source.

S1. Zhang Y, Luo Y, Pendry JB *et al.* Transformation-Invariant Metamaterials. *Phys Rev Lett* 2019; **123**: 067701.
